# Deadline extended: *Eurosurveillance* is looking for a seconded national expert in 2026

**DOI:** 10.2807/1560-7917.ES.2025.30.45.202511137

**Published:** 2025-11-13

**Authors:** Eurosurveillance editorial team

**Affiliations:** 1 European Centre for Disease Prevention and Control (ECDC), Stockholm, Sweden

The *Eurosurveillance* editorial team welcomes applications based on the current call for seconded national experts launched by the journal’s publisher, the European Centre for Disease Prevention and Control (ECDC).

Established in 1996, *Eurosurveillance* is one of the leading infectious disease journals with a strong history of public health impact. The small and enthusiastic team is looking for a colleague with interest and experience in infectious disease epidemiology, prevention and control and science communication/translation to support evidence-based public health action and decision-making.

If you are also proficient in critically appraising the quality of scientific articles, editing scientific texts together with a strong interest in publication ethics to strengthen trust in science, please send us your application.

The deadline has been extended to **5 December 2025.**

For more details on roles, responsibilities and requirements consult https://www.ecdc.europa.eu/sites/default/files/documents/call-seconded-national-experts-2025.pdf

